# Risk factors for hypotension in anaesthetized dogs: a retrospective analysis of 390 cases

**DOI:** 10.3389/fvets.2026.1846419

**Published:** 2026-06-19

**Authors:** Benedetta Mignini, Albane Rives, Massimiliano Degani, Chiara Talarico, Frédéric Farnir, Charlotte Sandersen

**Affiliations:** 1 Veterinary Anaesthesiology, Companion Animal and Equine Clinical Department, Faculty of Veterinary Medicine, University of Liège, Liège, Belgium; 2 Sustainable Productions, Veterinary Management of Animal Resources Department, Faculty of Veterinary Medicine, University of Liège, Liège, Belgium

**Keywords:** anaesthesia, blood pressure, complication, dog, hypotension

## Abstract

**Introduction:**

Hypotension during anaesthesia is related to potential adverse outcomes in human and veterinary patients. Careful monitoring of arterial blood pressure during anaesthesia is crucial. This retrospective study aimed at identifying risk factors for hypotension in anaesthetized dogs.

**Methods:**

Records from general anaesthetics in dogs between October 2023 and April 2024 were reviewed. Data regarding dog signalment, American Society of Anaesthesiologists (ASA) classification, procedure, employed drugs, mechanical ventilation, regional anaesthesia, fluid therapy, duration of anaesthesia, procedures, technique of arterial blood pressure monitoring, were gathered. Primary outcome was the development of hypotension: Mean Arterial Pressure (MAP) ≤ 60 mmHg for at least 3 consecutive measurements 5 min apart. Data were analyzed with descriptive statistics and multivariable logistic regression, the Mann Whitney *U* test and tests of linearity on continuous variables. Internal validation of the model and power estimation were performed.

**Results:**

Statistical significance was set at *p* < 0.05. A total of 390 records were analyzed. Hypotension was present in 72/390 cases (18.5%). An increased Odds Ratio (OR) for hypotension was found with abdominal surgery (overall OR 5.56, 95% CI 2.03–16.00, *p* = 0.001; abdominal versus (vs) diagnostic OR 5.56, 95% CI 1.33–23.2, *p* = 0.009; abdominal vs. orthopaedic OR 5.28, 95% CI 1.46–19.1, *p* = 0.004), age <12 months (overall OR 3.12, 95% CI 1.30–7.42, *p* = 0.010; young vs. adult OR 3.12, 95% CI 1.11–8.81, *p* = 0.027) and increasing duration of anaesthesia (OR 1.01, 95% CI 1.00–1.01, *p* < 0.001). OR for hypotension decreased with higher body mass (OR 0.96, 95% CI 0.94–0.98, *p* = 0.001), use of regional anaesthesia (overall for epidural-spinal OR 0.14, 95% CI 0.02–0.57, *p* = 0.014; overall for nerve blocks OR 0.24, 95% CI 0.11–0.50, *p* < 0.001; epidural-spinal vs. no regional anaesthesia OR 0.14, 95% CI 0.02–0.91, *p* = 0.036; nerve block vs. no regional anaesthesia OR 0.24, 95% CI 0.09–0.60, *p* < 0.001), and mechanical ventilation (overall OR 0.52, 95% CI 0.27–0.96, *p* = 0.039; mechanical ventilation vs. no mechanical ventilation OR 0.52, 95% CI 0.27–0.97, *p* = 0.039).

**Discussion:**

Identification of risk factors for hypotension in anaesthetized dogs can contribute to developing prevention and treatment strategies to improve outcomes and safety.

## 1 Introduction

Maintenance of oxygen delivery to tissues is one of the aims of safe anaesthetic practice (1). Oxygen delivery and blood flow are influenced by arterial oxygen content, cardiac output, vascular resistance, and mean arterial blood pressure (MAP), all of which can be negatively affected by general anaesthesia. Therefore, optimizing cardiovascular function in anaesthetized patients is crucial.

Under most clinical conditions, it is not possible to measure cardiac output and calculate systemic vascular resistance during general anaesthesia; on the other hand, arterial blood pressure (ABP) can be easily measured. ABP monitoring during anaesthesia serves as a critical surrogate indicator of perfusion and cardiac output (2). Hypotension during anaesthesia can result in tissue hypoperfusion, disruption of autoregulation and potential organ damage. This can be especially detrimental to organs that have high oxygen demand and tight autoregulation of blood flow, such as the brain, heart and kidneys (3).

In human medicine, the definition of hypotension in anaesthetized patients is highly debated, incorporating values of Systolic Arterial Pressure (SAP) and/or MAP, absolute or relative thresholds, various indications of duration and severity (4–7). Although different definitions affect its reported frequency and association with adverse outcomes (4–6), studies have identified an association between intraoperative hypotension and acute kidney injury (7, 8), myocardial injury (7–9), cerebrovascular accidents, and mortality (8).

Hypotension during anaesthesia has various definitions also in veterinary medicine (10, 11). However, MAP lower than 60 mmHg is commonly considered as hypotension in anaesthetized dogs (12–19). SAP is also inconsistently included in the definition (12, 13, 15, 17, 18), or is used as the only threshold value (20).

Despite pointing out the lack of evidence in veterinary literature to support this definition of hypotension, a survey among Diplomates of the American and European Colleges of Veterinary Anaesthesia and Analgesia found good agreement among specialists to define hypotension in anaesthetized dogs as MAP < 62 ± 4 (mean ± standard deviation) mmHg; respondents also agreed in considering SAP < 87 ± 8 (mean ± standard deviation) mmHg and SAP < 87 ± 6 (mean ± standard deviation) as hypotension in surgical and diagnostic cases, respectively (21).

Evidence on hypotension during anaesthesia in dogs is limited. The reported prevalence is variable (12–14, 16–18). It has been associated with an increased risk of postoperative mortality (16, 20), septic peritonitis after digestive tract surgery (22), potential acute kidney injury (23, 24), small intestinal dehiscence (15), adverse events after ventral slot decompression (persistent or worsening neck pain, haemorrhage, need for postoperative mechanical ventilation) (25). Other studies did not identify associations with postoperative complications (17, 18). Preanaesthetic urinary specific gravity has been evaluated in dogs as an indicator of subclinical dehydration and a potential risk factor for hypotension during anaesthesia, with conflicting results (26, 27), while an increased risk of hypotension during anaesthesia has been found in dogs with lower body mass (19).

After a comprehensive literature search for keywords “anaesthesia/anesthesia,” “hypotension-intraoperative,” “hypotension-perioperative” and “dog,” published from 1995 to 2026, we found that studies exploring a broad range of risk factors for hypotension in anaesthetized dogs are lacking. A limited number of studies in the search investigated risk factors for perioperative hypotension in dogs (19, 26, 27). The aim of this retrospective analysis was to identify potential pre- and intraoperative risk factors and protective factors associated with hypotension during anaesthesia in dogs.

## 2 Materials and methods

### 2.1 Study design

This retrospective cohort study is reported in accordance with the veterinary extension of the STrengthening the Reporting of OBservational Studies in Epidemiology—STROBE VET Statement (28). Due to the retrospective observational nature of the study, ethical approval was not required.

### 2.2 Data collection and management

Anaesthetic records from dogs undergoing general anaesthesia for diagnostic and surgical procedures at the Companion Animals section of the Veterinary Teaching Hospital of the University of Liège were collected from 1 October 2023 to 30 April 2024. The sampling period was fixed at 7 months due to logistical and administrative constraints. Informed consent for the use of clinical records in an anonymized format, including anaesthetic records, was obtained for each patient at admission as per standard hospital procedure. Anaesthetics were performed by residents with supervision from board-certified anesthesiologists. Standard clinical monitoring was used, including: electrocardiography, pulse oximetry, capnography, arterial blood pressure measurement with invasive technique or non-invasively with oscillometry at the discretion of the resident managing the case, expired halogenated agents and spirometry when appropriate monitoring was available. Invasive blood pressure measurement was accomplished with an arterial catheter placed in the dorsal pedal artery, connected via a noncompliant tubing to a pressure transducer from the monitor. The transducer was placed at the level of the right atrium and zeroed to atmospheric pressure. The transducer-tubing-catheter assembly was continuously flushed with heparinized saline in a pressurized bag. Non-invasive blood pressure monitoring with oscillometry employed an inflatable cuff of appropriate size (width of the cuff approximately 30–40% of limb circumference) placed around the antebrachium. Cyclic inflation and deflation of the cuff to obtain ABP measurements was automatically set at 3-min intervals (3). Monitors employed in the study were Mindray PM-8000, PM-9000, and ePM 12 Vet (Shenzhen Mindray Bio-Medical Electronics Co., Ltd., Shenzhen, China). Monitored variables were recorded every 5 min. If both invasive and non-invasive blood pressure measurement techniques were employed in the same dog, then invasive values were considered.

General anaesthesia lasting < 20 min, an incomplete anaesthetic record (variables not recorded for ≥ 10 min and/or missing information regarding signalment and procedure), and lack of MAP measurement during anaesthesia (i.e., dogs equipped with a Doppler monitor), were considered as exclusion criteria. A case-oriented approach was applied (i.e., for dogs undergoing more than one general anaesthetic in the recruitment period, every anaesthetic was considered as a separate case).

Data from anaesthetic records were entered into a table (Excel, Microsoft Corporation, WA, USA). Entered data included age (months), weight (kilograms), breed group according to the Fédération Cynologique Internationale breed nomenclature (29), including an additional group for mixed breed dogs, sex and neutering status, Body Condition Score (BCS, 1–9), ASA physical status classification (I–V), if the procedure was an emergency, type of procedure, drugs employed in premedication, induction and maintenance of anaesthesia, use of mechanical ventilation (yes/no), presence and type of regional anaesthesia techniques (no regional anaesthesia, epidural/spinal, nerve blocks; both perineural and fascial plane blocks were included in the “nerve block” group to increase group size), type of intravenous fluids used (crystalloid or blood products) and the duration (minutes) of general anaesthesia (from induction to discontinuation of maintenance agents), surgery (from skin incision to completion of skin closure) or diagnostic procedure (from start to finish of image acquisition). To facilitate analysis, age and BCS were further classified, respectively, into “young” (< 12 months), “adult” (13–96 months), “senior” (>96 months), and “underweight” (BCS 1–3), “average” (BCS 4–6), and “overweight/obese” (BCS 7–9). Procedure type was classified as follows: diagnostic; radiography, endoscopy including foreign body removal via endoscopy, computed tomography, magnetic resonance imaging, cerebrospinal fluid collection, fine-needle aspirates and biopsies. Minor; procedures not involving the opening of a body cavity, skin sutures and flaps, mastectomy, orchiectomy, ophthalmic surgeries, central venous catheter placement. Orthopaedic-neurologic; osteosynthesis, arthroscopy, dorsal hemilaminectomy, ventral slot decompression. Abdominal; surgical opening of the abdomen, including laparoscopy. Thoracic; opening of the thorax and procedures on intrathoracic structures, including diaphragmatic hernia repair and interventional cardiology. When diagnostic procedures were followed by surgery during the same anaesthetic (e.g., spine computed tomography followed by dorsal hemilaminectomy for intervertebral disk disease), classification was according to type of surgery.

Employed drugs were classified as follows: premedication drugs; sedative-tranquillizers (none, alpha2 agonists, acepromazine), opioids (none, full *μ* agonists, agonists/antagonists), adjunctive drugs (none, midazolam, ketamine, lidocaine, midazolam + ketamine). Induction drugs; hypnotic agent (propofol, alfaxalone), adjunctive drugs (none, midazolam, ketamine, lidocaine, midazolam + ketamine, other). Maintenance drugs; maintenance agent (isoflurane, propofol), alpha2 agonist constant rate infusion (CRI; yes, no), adjunctive CRI (none, ketamine, opioids, lidocaine, ketamine + opioids, other). Recorded variables are listed in Table 1.

**Table 1:** Demographic data from 390 records of dogs undergoing general anaesthesia in a University teaching hospital.

Variable	Median (IQR, range) or *n* (%)
Age in months	86.5 (45–119, 3–191)
Age group	
Young	37 (9.5)
Adult	197 (50.5)
Senior	156 (40)
Weight in kg	20.75 (10–33.5, 1.46–76)
Breed	
Sheepdogs–Cattledogs	60 (15.4)
Pinscher–Schnauzer–Molossoid	50 (12.8)
Terriers	48 (12.3)
Retrievers–Flushing dogs	45 (11.5)
Companion–Toy	80 (20.5)
Other	69 (17.7)
Mixed breed	38 (9.7)
Sex	
Male neutered	125 (32)
Male intact	89 (23)
Female neutered	114 (29)
Female intact	62 (16)
BCS	
3	14 (3.6)
4	64 (16.4)
5	154 (39.5)
6	92 (23.6)
7	47 (12)
8	10 (2.6)
Other	9 (2.3)
Weight group	
Average	310 (80)
Overweight	63 (16)
Underweight	17 (4)
Premedication	
Alpha2 agonists + full μ opioids	206 (53)
Full μ opioids	109 (28)
Alpha2 agonists + full μ opioids + ketamine	24 (6)
Alpha2 agonists + agonist – antagonist opioids	17 (4)
Agonist–antagonist opioids	8 (2)
Other	26 (7)
Induction	
Propofol	177 (45.4)
Propofol + midazolam	147 (37.7)
Propofol + ketamine	38 (9.7)
Propofol + midazolam + ketamine	11 (3)
Other	17 (4)
Maintenance	
Isoflurane	171 (43.8)
Isoflurane + alpha2 CRI	68 (17.4)
Isoflurane + opioid CRI	43 (11)
Isoflurane + alpha2 CRI + opioid CRI	25 (6.4)
Isoflurane + alpha2 CRI + ketamine CRI	22 (5.6)
Isoflurane + opioid CRI + ketamine CRI	19 (5)
Other	42 (10.8)
Regional anaesthesia	
No	242 (62)
Nerve block	126 (32.3)
Epidural–spinal	22 (5.6)
Mechanical ventilation	
Yes	191 (47)
No	199 (53)
ASA	
2	178 (46)
3	145 (37.2)
4	40 (10)
1	20 (5)
5	7 (1.8)
Emergency	
No	287 (73.6)
Yes	103 (26.4)
Type of procedure	
Diagnostic	87 (22)
Minor	135 (35)
Orthopaedic–neurosurgery	84 (21.5)
Abdominal	80 (20.5)
Thoracic	4 (1)
Duration of anaesthesia	117.5 (70–165, 20–485)
Duration of surgery	65 (43–91, 10–330)
Type of fluids	
Crystalloids	339 (87)
No fluids	36 (9.2)
Blood products	15 (3.8)

Data are expressed as median (interquartile range-IQR) or n (%) where appropriate. Constant rate infusion (CRI).

The primary outcome was the development of at least one episode of hypotension. Hypotension was defined using previously reported thresholds as MAP ≤ 60 mmHg (12–19, 22, 24–27) for at least three (3) consecutive measurements 5 min apart, i.e., hypotension lasting ≥ 10 min. These criteria were adopted to avoid classifying artefactual readings, which are usually single readings, as hypotension (4, 14). Preoperative and intraoperative characteristics were considered as explanatory variables.

### 2.3 Statistical analysis

Statistical analysis was performed with freely available software (R version 4.4.3, The R Foundation for Statistical Computing, Austria).

Initially, descriptive statistics were obtained; results are expressed as numbers and percentages or median and interquartile range, as appropriate. Subsequently, all candidate explanatory variables were included in a multivariable logistic regression model. A bidirectional stepwise selection of explanatory variables based on the Akaike Information Criterion was then applied, and the final model with the lowest Akaike Information Criterion value obtained was fitted using the retained variables. Multicollinearity was checked by calculating the Variance Inflation Factor for each retained variable. After multivariable logistic regression with standard reference values, a pairwise comparison using Tukey-adjusted contrasts was conducted to compare all possible pairs of levels within each categorical variable. Odds Ratios (OR) and OR 95% Confidence Intervals (95% CI) were calculated from the final model. Significance was set at *p* < 0.05.

Due to the exploratory and retrospective nature of the study, a formal prospective power calculation was not performed. However, as described in the literature, a “partial *post hoc*” power estimation was provided, as it is considered a more appropriate approach than simple post-hoc power assessment (30). It was performed using a Monte Carlo simulation using univariable logistic regression and a range of different effect sizes, cross-compared with the observed OR for each variable included in the final multivariable model, since collinearity was low and the univariable models were similar to the multivariable model.

Continuous variables retained in the final model were compared between hypotensive and non-hypotensive cases with the Mann–Whitney *U* test. Linearity of retained continuous variables was assessed by comparison of the final multivariable logistic regression model with a splined model with 3 degrees of freedom for each continuous variable using the Likelihood Ratio Test.

Internal validity of the model was assessed by calculating the Event Per Variable (EPV) value and with internal bootstrap analysis, taking 1,000 bootstrap samples and refitting the model, using a penalized ridge regression to improve convergence.

## 3 Results

A total of 448 anaesthetic records were initially collected, and 58 were excluded: 29 general anaesthetics lasting less than 20 min, 10 incomplete records, and 19 records without invasive or oscillometric blood pressure measurement during anaesthesia. The remaining 390 records for 332 dogs were included in the analysis. Of the 332 dogs, 304/332 had 1 anaesthesia (91.6%), 19/332 had 2 anaesthesias (5.7%), 4/332 had 3 anaesthesias (1.2%), 2/332 had 4 anaesthesias (0.6%), and 3/332 had ≥ 5 anaesthesias (0.9%). ABP was measured with oscillometry in 301/390 anaesthetics (77%), while invasive blood pressure was used in 89/390 cases (23%). Hypotension was present in 72/390 cases (18.5%). Oscillometry was used in 49/72 (68%) of the hypotensive cases, and the remaining 23/72 (32%) had invasive blood pressure monitoring. Demographic data are summarized in Table 1. For each variable, when ≥2 categories contained < 20 individuals each, these were grouped together as “other.”

After the bidirectional stepwise selection of explanatory variables, the final model included age group, body mass, regional anaesthesia, mechanical ventilation, procedure type, and duration of anaesthesia. Variance Inflation Factor was < 5 for all variables, indicating weak collinearity, so they were all retained in the model (Tables 2, 3).

**Table 2:** Odds Ratios (OR), 95% Confidence Intervals (95% CI), and *p* values for the explanatory variables for perianaesthetic hypotension in 390 records of dogs undergoing general anaesthesia in a University teaching hospital that were included in the multivariable logistic regression model with standard reference values.

Variable	OR	95% CI	*p* value
Age group			
Adult	Referent	N/A	N/A
Senior	1.21	0.64–2.31	0.600
Young	3.12	1.30–7.42	0.010
Weight	0.96	0.94–0.98	0.001
Regional anaesthesia			
No Regional anaesthesia	Referent	N/A	N/A
Epidural-spinal	0.14	0.02–0.57	0.014
Nerve block	0.24	0.11–0.50	<0.001
Mechanical ventilation			
No	Referent	N/A	N/A
Yes	0.52	0.27–0.96	0.039
Procedure type			
Diagnostic	Referent	N/A	N/A
Minor	2.12	0.90–5.26	0.094
Orthopaedic-neurosurgery	1.05	0.34–3.19	>0.900
Abdominal	5.56	2.03–16.00	0.001
Thoracic	1.18	0.05–11.9	0.900
Duration of anaesthesia	1.01	1.00–1.01	<0.001

Reference category for each variable is the first category.

**Table 3:** Odds Ratios (OR), 95% Confidence Intervals (95% CI), and *p* values for the explanatory variables for perianaesthetic hypotension in 390 records of dogs undergoing general anaesthesia in a University teaching hospital that were included in the multivariable logistic regression model, after pairwise comparisons with Tukey-adjusted contrasts of all possible couples of categories within each variable.

Variable	OR	95% CI	*p* value
Age group			
Senior vs. adult	1.21	0.56–2.62	0.800
Young vs. adult	3.12	1.11–8.81	0.027
Young vs. senior	2.57	0.89–7.40	0.091
Weight	0.96	0.94–0.98	0.001
Regional anaesthesia			
Epidural-spinal vs. no regional anaesthesia	0.14	0.02–0.91	0.036
Nerve block vs. no regional anaesthesia	0.24	0.09–0.60	<0.001
Nerve block vs. epidural-spinal	1.75	0.25–12.3	0.800
Mechanical ventilation			
Yes vs. no	0.52	0.27–0.97	0.039
Procedure type			
Minor vs. diagnostic	2.12	0.62–7.17	0.400
Orthopaedic-neurosurgery vs. diagnostic	1.05	0.23–4.90	>0.900
Orthopaedic-neurosurgery vs. minor	0.50	0.14–1.73	0.500
Abdominal vs. diagnostic	5.56	1.33–23.2	0.009
Abdominal vs. minor	2.63	0.86–7.98	0.120
Abdominal vs. orthopaedic-neurosurgery	5.28	1.46–19.1	0.004
Thoracic vs. diagnostic	1.18	0.04–37.5	>0.900
Thoracic vs. minor	0.56	0.02–15.7	>0.900
Thoracic vs. orthopaedic-neurosurgery	1.12	0.04–34.2	>0.900
Thoracic vs. abdominal	0.21	0.01–6.49	0.700
Duration of anaesthesia	1.01	1.00–1.01	<0.001

vs, Versus.

Age < 12 months, abdominal surgery, and increasing duration of anaesthesia were significantly correlated with increased OR of hypotension during anaesthesia, while increasing body mass, the use of regional anaesthesia and mechanical ventilation were linked to a decreased OR of hypotension (Table 2). Furthermore, dogs undergoing abdominal surgery showed a significantly higher OR of hypotension when compared to those submitted for diagnostic or orthopaedic procedures and dogs aged < 12 months had a significantly higher OR of hypotension than adult dogs (Table 3).

The Mann–Whitney *U* test applied to continuous variables identified a significant difference in duration of anaesthesia (*W* = 14,028, *p* = 0.002814) and body mass (*W* = 8,817, *p* = 0.002322) between hypotensive and non-hypotensive dogs.

Comparison between linear and splined regression models with the Likelihood Ratio Test showed non-significant results (*p* = 0.1378 for body mass, *p* = 0.1784 for duration of anaesthesia), confirming a linear relationship between continuous variables and OR of hypotension. A linear relationship means that for each unit increase of anaesthesia duration and body mass the OR of hypotension will also increase according to the relationship OR^X^, where X is the duration of anaesthesia in minutes or body mass in kilograms. Specifically, for every 1 min increase in anaesthesia duration, there was a 1% increase in the OR of hypotension (OR 1.01, 95% CI 1.00–1.01, *p* < 0.001), whereas for every 1 kilogram increase in body mass OR of hypotension decreased 4% (OR 0.96, 95% CI 0.94–0.98, *p* = 0.001).

EPV value was 12. This meets the minimum threshold of 10 events per variable recommended for logistic regression modelling (31), though it falls below the more conservative criterion of 15 advocated in recent literature (32). Model stability was therefore further evaluated using internal bootstrap validation (32). Internal bootstrap analysis showed acceptable stability of the model, with satisfactory discriminative power to detect hypotensive cases despite a moderate risk of overfitting (optimism-corrected values: Area Under Discrimination Curve 0.7377; *R*^2^ value 0,1,682; intercept calibration - 0.0805; calibration slope 0.9317; Brier score 0.1332).

Partial *post hoc* power estimation yielded variable results, with wide 95% CI, precluding a definitive estimation of statistical power. Results are reported in Supplementary Table 1.

## 4 Discussion

This retrospective cohort study analyzed factors related to hypotension in dogs undergoing general anaesthesia for a variety of procedures.

Prevalence of hypotension in the present study is within the range reported in the literature. However, this range is wide for dogs: from 7 to 92% (10, 12–14, 16, 18, 19, 26). This variability might reflect different populations, monitoring techniques, and inconsistent definitions of hypotension during anaesthesia. Variable thresholds, values of interest (SAP or MAP or both), measurement techniques (invasive and/or noninvasive), duration of hypotensive episodes (number of measurements, minutes, or no indication of duration), render evidence on this subject confusing and difficult to compare. Prevalence of hypotension during anaesthesia reported in the present study is at the lower end of the published range. The strict definition of hypotension employed might contribute to this finding. In human medicine, studies have shown that different definitions of intraoperative hypotension have a significant effect on reported frequency and association with adverse outcomes (4–6). Speculatively, this may also apply to veterinary medicine, but evidence is lacking. Furthermore, it was not possible to standardize arterial blood pressure measurement techniques in our clinical study. Invasive blood pressure measurement and non-invasive oscillometric techniques were both used in the cohort, and since oscillometry can be less accurate than invasive blood pressure monitoring (3), an inaccurate estimation of hypotension due to different monitoring techniques cannot be ruled out.

In the present study, the use of both neuraxial anaesthesia and nerve blocks had a decreased OR for hypotension (for epidural-spinal, OR 0.14, 95% CI 0.02–0.57, *p* = 0.014; for nerve blocks, OR 0.24, 95% CI 0.11–0.50, *p* < 0.001 in the multivariable logistic regression with prespecified standard reference values; OR 0.14, 95% CI 0.02–0.91, *p* = 0.036 for epidural-spinal vs. no regional anaesthesia; OR 0.24, 95% CI 0.09–0.60, *p* < 0.001 for nerve block vs. no regional anaesthesia). The narrow 95% CI suggests a precise estimation of effect size. This finding is of particular interest since it can be controlled by the anaesthetist. This result is in accordance with human and veterinary evidence showing a reduction in inhalant anaesthetic use concurrent with the use of epidural anaesthesia (33–35) and peripheral nerve blocks (35, 36). This reduction minimizes cardiovascular depression from inhalant anesthetics (37, 38). Possible suggested mechanisms for this finding include a decrease in afferent stimuli reaching the central nervous system and effective analgesia due to epidural anaesthesia and peripheral nerve blocks, reducing central nervous system (33) and hypothalamus-pituitary–adrenal axis activation (35). Regional anaesthetic techniques have also been associated with a significantly decreased risk of perianaesthetic mortality in dogs, owing to reduced hypnotic doses, cardiovascular and respiratory stability, and a reduction of perioperative stress (39).

Mechanical ventilation was associated with decreased OR for hypotension (OR 0.52, 95% CI 0.27–0.96, *p* = 0.039 in the multivariable logistic regression with prespecified standard reference values; OR 0.52, 95% CI 0.27–0.97, *p* = 0.039 for mechanical ventilation vs. no mechanical ventilation). The non-linearity of OR and 95% CI for values between 0 and 1 results in an estimation of effect size that

can change across values of the predictor. Heart-lung interactions are a complex phenomenon, and current evidence suggests that mechanical ventilation and positive end expiratory pressure can have a beneficial effect on left ventricular (LV) function with a decrease in LV afterload and improved output in patients with LV systolic dysfunction (40–42). In healthy patients, on the other hand, mechanical ventilation and positive end expiratory pressure have detrimental effects on LV haemodynamics (40). Heart-lung interactions in mechanically ventilated dogs are also the basis for various fluid responsiveness indices that are used to guide fluid therapy during anaesthesia. These indices show moderate to good predictive capacity for fluid responsiveness (43). However, the protective potential of mechanical ventilation against hypotension found in our cohort could represent residual confounding, related to a larger use of mechanical ventilation in animals that are haemodynamically stable and thus less prone to hypotension, rather than a real protective effect. Moreover, since information on ventilator settings and expired CO2 values was not included in the analysis, the potential haemodynamic impact of these factors cannot be quantified.

In the present study, a significant difference has been found in body mass between hypotensive and non-hypotensive cases. Furthermore, a lower OR of anaesthesia-related hypotension corresponded to an increase in body mass of the dogs. The narrow 95% CI suggests a precise estimation of effect size. The linear relationship with additive effect shown between OR and unit increase means that for every 1 kg increase in body mass from the minimal value of 1 kg the OR of hypotension during anaesthesia decreases 4% (OR 0.96, 95% CI 0.94–0.98, *p* = 0.001). The relationship is true in the observed range of values (Table 1), which reflects a large range of body masses that can be commonly encountered in clinical practice. This result is in line with published evidence (14, 19, 44). It is difficult to determine if smaller individuals develop hypotension during anaesthesia more often than dogs with higher body mass, or if this reflects the fact that it is more technically challenging to obtain reliable ABP readings in smaller dogs, using invasive or noninvasive methods (19). The difficulty in placing arterial catheters for invasive blood pressure measurement, as well as in choosing appropriately sized cuffs and minimizing positional artifacts in dogs with lower body mass is considerable. Furthermore, smaller-sized individuals might be prone to relative drug overdose (14, 44) and hypothermia (44), both of which can precipitate hypotension.

Abdominal surgery was significantly associated with increased risk of hypotension, in comparison with diagnostic and orthopaedic procedures (OR 5.56, 95% CI 2.03–16.00, *p* = 0.001 in the multivariable logistic regression with prespecified standard reference values; OR 5.56, 95% CI 1.33–23.2, *p* = 0.009 for abdominal versus (vs) diagnostic; OR 5.28, 95% CI 1.46–19.1, *p* = 0.004 for abdominal vs. orthopaedic). The wide OR 95% CI does not allow for a precise estimation of effect size (Tables 2, 3). Human and veterinary evidence suggests that patients undergoing laparotomy may be affected by absolute hypovolemia from extensive peritoneal effusion (45), fluid losses in the gastrointestinal tract, diarrhoea and vomiting, sequestration of fluids in the gastrointestinal lumen and intraabdominal haemorrhage, or relative hypovolemia from sepsis-induced vasodilation (46). Mesenteric traction syndrome could also contribute to hypotension during abdominal surgery. This syndrome, where hypotension, tachycardia and peripheral flushing result from prostacyclin release during surgical manipulation of the mesentery, is described in humans and pigs (47, 48). We cannot exclude that a similar condition is present in dogs. The mechanisms presented above can favor the development of hypotension during abdominal surgery and can contribute to explaining our findings.

A significant difference was identified in duration of anaesthesia between hypotensive and non-hypotensive dogs. Furthermore, increasing duration of anaesthesia was associated with increased OR of hypotension in this cohort. The narrow 95% CI suggests a precise estimation of effect size. The linear relationship with additive effect found between OR and unit increase means that for every 1 min increase of anaesthetic duration from the minimal value of 20 min (as established in the inclusion and exclusion criteria) the OR of hypotension increases 1% (OR 1.01, 95% CI 1.00–1.01, *p* < 0.001). The relationship is true in the observed range of values (Table 1), which reflects a large range of anaesthetic durations that can be commonly encountered in clinical practice. Increasing duration of anaesthesia is an established risk factor for perianaesthetic mortality in small animals and horses (44, 49). On the other hand, previous studies did not identify an association between anaesthetic duration and an increased prevalence of hypotension (19). Nevertheless, a longer anaesthetic might reflect more profound cardiovascular depression and physiological derangement, and also complex and unstable cases that are more prone to developing hypotension during anaesthesia (44). Furthermore, the increased tendency to hypothermia in longer anaesthetics might contribute to precipitate hypotension, as previously reported (19).

Age <12 months had a significant association with increased OR of hypotension during anaesthesia (OR 3.12, 95% CI 1.30–7.42, *p* = 0.010, in the multivariable logistic regression with prespecified standard reference values; OR 3.12, 95% CI 1.11–8.81, *p* = 0.027 for young vs. adult). The wide OR 95% CI does not allow for a precise estimation of effect size (Tables 2, 3). This finding is in agreement with previous evidence, where an increased prevalence of hypotension during anaesthesia in young dogs has been possibly linked to their cardiovascular immaturity (14). Paediatric patients also have an increased perianaesthetic mortality (39). On the other hand, paediatric dogs have lower MAP than adults, due to reduced vascular resistance that increases gradually until 9 months of age (50), so the definition of hypotension used in the present study might not be completely applicable to younger dogs.

This study has the following limitations. First, data were collected and analyzed retrospectively, and ABP was measured with invasive and noninvasive techniques at the discretion of the clinician in charge of the case. In the majority of cases in the present study, ABP was measured with oscillometry, and the impact of different measurement techniques was not assessed. Noninvasive techniques are less accurate than invasive measurement of ABP (3), so an inaccurate estimation of hypotension prevalence in this cohort is possible. Second, the cohort was relatively small and there was a limited number of cases for certain variables. This limits the statistical power, therefore results have to be interpreted cautiously and it is possible that other factors related to hypotension in anaesthetized dogs with small effect sizes remained undetected. On the other hand, satisfactory internal validity of the model demonstrated by internal bootstrapping and EPV supports our findings. Third, although every effort was made to collect all anaesthetic records in the study period, it is possible that some anaesthetic records were not included because they were not handed over for analysis from participants for various reasons, reflecting a potential for selection bias. Fourth, other factors with a potential impact on haemodynamics during anaesthesia, such as the occurrence of hypothermia and bradycardia, the contribution of estimated blood loss to hypovolemia, the use of vasoactive drugs, ventilator settings, expired CO2, and expired halogenated agents were not included in the analysis. Moreover, the effect of different inhalant agents or total intravenous anaesthesia for maintenance could not be assessed since isoflurane was employed in the vast majority of cases. Finally, prevalence of hypotension in the present cohort might have been higher using a less strict definition, for example eliminating a minimal duration threshold. This is a common issue in the assessment of perioperative hypotension, and studies in human medicine demonstrated that stringent definitions of hypotension correspond to lower incidence (5). Nevertheless, we selected a MAP threshold that was already present in the literature for consistency with published work (12–19), and a minimal duration limit of hypotension was set to detect hypotensive cases and exclude accidental artefactual values (4, 14).

Identifying risk factors is the first step to implement strategies to prevent and promptly treat hypotension during anaesthesia and improve patient safety, given the potential adverse outcomes associated with it (15, 16, 22–25).

Further research is needed to verify the generalisability of our results. It might include testing different definitions of hypotension and monitoring techniques, larger, multicentre studies on risk factors, patient- or procedure-specific risk factors, species-specific associations between hypotension and adverse postoperative outcomes, identify species- and organ-specific harm thresholds for hypotension during anaesthesia, and prospective studies on prevention and treatment interventions.

Despite limitations, the present analysis provides information on potential risk factors and protective factors for hypotension during anaesthesia in dogs undergoing a range of clinical procedures. Specifically, age <12 months, abdominal surgery and increasing anaesthesia duration were identified as risk factors for hypotension during anaesthesia, whereas increasing body mass and the use of regional anaesthesia and mechanical ventilation emerged as protective factors. Factors that can be controlled by the anaesthetist, such as the use of regional anaesthesia, can be of particular interest in patient management, while other patient- and procedure-specific aspects such as age, body mass and procedure type warrant increased vigilance for complications but cannot be changed. Minimizing the duration of anaesthesia should be part of a joint effort from the anaesthetic and surgical team through appropriate planning and communication throughout the procedure. The present study also highlights the lack of evidence on hypotension in anaesthetized dogs, from definition and measurement issues to limited information on its possible association with adverse outcomes. Further research is needed to improve our understanding on hypotension during anaesthesia in dogs.

## Data Availability

The original contributions presented in the study are included in the article/Supplementary material, further inquiries can be directed to the corresponding author.
