# Supplementary material for: Risk factors for hypotension in anaesthetized dogs: a retrospective analysis of 390 cases
Source: Front Vet Sci. 2026 Jun 19;13:1846419. doi: 10.3389/fvets.2026.1846419 (PMC13327969; doi:10.3389/fvets.2026.1846419)
Supplement: Supplementary file 1 [file Table_1.DOCX]

**Supplementary Table 1.** Univariable regression power estimation, calculated with a Monte Carlo simulation and multiple effect sizes, cross-compared with observed Odds Ratios (OR) of explanatory variables for hypotension in 390 dogs undergoing general anaesthesia in a University teaching hospital: OR, Estimated power values and 95 % CI for OR and estimated power are reported.

| **Characteristic** | **OR** | **95% CI** | **Estimated Statistical Power** | **Estimated power 95 % CI** |
| --- | --- | --- | --- | --- |
| Age group: senior-geriatric vs adult | 1.21 | 0.56 - 2.62 | 0.16 | 0.063 – 0.953 |
| Age group: young vs adult | 3.12 | 1.11 – 8.81 | 0.985 | 0.133 – 1.000 |
| Age group: young vs senior-geriatric | 2.57 | 0.89 – 7.40 | 0.953 | 0.063 – 1.000 |
| Body mass | 0.96 | 0.94–0.98 | 0.065 | 0.053 – 0.065 |
| Epidural-spinal vs no regional anaesthesia | 0.14 | 0.02 - 0.91 | 1 | 0.075 – 1.000 |
| Nerve block vs no regional anaesthesia | 0.24 | 0.09 - 0.60 | 1 | 0.383 – 1.000 |
| Nerve block vs epidural-spinal | 1.75 | 0.25 – 12.3 | 0.530 | 0.045 – 1.000 |
| Mechanical ventilation: yes vs no | 0.52 | 0.27 – 0.97 | 0.720 | 0.028 – 1.000 |
| Procedure type: minor vs diagnostic | 2.12 | 0.62 – 7.17 | 0.678 | 0.110 – 0.978 |
| Procedure type: Orthopaedic-neurosurgery vs diagnostic | 1.05 | 0.23 - 4.90 | 0.113 | 0.110 – 1.000 |
| Procedure type: orthopaedic-neurosurgery vs minor | 0.50 | 0.14 - 1.73 | 0.708 | 0.110 – 1.000 |
| Procedure type: abdominal vs diagnostic | 5.56 | 1.33 – 23.2 | 0.985 | 0.230 – 0.985 |
| Procedure type: abdominal vs minor | 2.63 | 0.86 - 7.98 | 0.835 | 0.110 – 0.985 |
| Procedure type: abdominal vs orthopaedic-neurosurgery | 5.28 | 1.46-19.1 | 0,985 | 0.360 – 0.985 |
| Procedure type: thoracic vs diagnostic | 1.18 | 0.04 - 37.5 | 0.173 | 0.110 – 1.000 |
| Procedure type: thoracic vs minor | 0.56 | 0.02-15.7 | 0.600 | 0.110 – 1.000 |
| Procedure type: thoracic vs orthopaedic-neurosurgery | 1.12 | 0.04-34.2 | 0.110 | 0.110 – 1.000 |
| Procedure type: thoracic vs abdominal | 0.21 | 0.01 - 6.49 | 1.000 | 0.110 – 1.000 |
| Duration of anaesthesia | 1.01 | 1.00 - 1.01 | 0.038 | 0.038-0,050 |
